# A New Method Based on Time-Varying Filtering Intrinsic Time-Scale Decomposition and General Refined Composite Multiscale Sample Entropy for Rolling-Bearing Feature Extraction

**DOI:** 10.3390/e23040451

**Published:** 2021-04-11

**Authors:** Jianpeng Ma, Song Han, Chengwei Li, Liwei Zhan, Guang-zhu Zhang

**Affiliations:** 1 School of Instrumentation Science and Engineering, Harbin Institute of Technology, Harbin 150001, China; 18B901017@stu.hit.edu.cn; 2 Aero Engine Corporation of China Harbin Bearing Co., Ltd., Harbin 150500, China; hs829325@163.com (S.H.); zhanliwei333@163.com (L.Z.); 3 Undergraduate College, Songsim Global Campus, The Catholic University of Korea, Bucheon-si 14662, Korea; zhangks@catholic.ac.kr

**Keywords:** rolling bearing, fault diagnosis, signal denoising, intrinsic time-scale decomposition, coyote optimization algorithm, generalized refined composite multiscale sample entropy

## Abstract

The early fault diagnosis of rolling bearings has always been a difficult problem due to the interference of strong noise. This paper proposes a new method of early fault diagnosis for rolling bearings with entropy participation. First, a new signal decomposition method is proposed in this paper: intrinsic time-scale decomposition based on time-varying filtering. It is introduced into the framework of complete ensemble intrinsic time-scale decomposition with adaptive noise (CEITDAN). Compared with traditional intrinsic time-scale decomposition, intrinsic time-scale decomposition based on time-varying filtering can improve frequency-separation performance. It has strong robustness in the presence of noise interference. However, decomposition parameters (the bandwidth threshold and B-spline order) have significant impacts on the decomposition results of this method, and they need to be artificially set. Aiming to address this problem, this paper proposes rolling-bearing fault diagnosis optimization based on an improved coyote optimization algorithm (COA). First, the minimal generalized refined composite multiscale sample entropy parameter was used as the objective function. Through the improved COA algorithm, optimal intrinsic time-scale decomposition parameters based on time-varying filtering that match the input signal are obtained. By analyzing generalized refined composite multiscale sample entropy (GRCMSE), whether the mode component is dominated by the fault signal is determined. The signal is reconstructed and decomposed again. Finally, the mode component with the highest energy in the central frequency band is selected for envelope spectrum variation for fault diagnosis. Lastly, simulated and experimental signals were used to verify the effectiveness of the proposed method.

## 1. Introduction

Rolling bearings are a key component in mechanical equipment. Their working status affects the operation of the entire equipment. The existence of bearing defects inevitably affects the dynamic performance of the equipment, and even damages the whole machine. Therefore, rolling-bearing fault monitoring and identification can allow for understanding the development of bearing faults. Maintenance strategies need to be developed in advance for minor faults that appear early, so as to avoid economic losses and accidents caused by equipment damage or sudden shutdowns [1,2,3]. Current research on bearing failures is mainly based on the analysis of vibration signals. Information that can characterize the running state of bearings is extracted from signals and through pattern-recognition algorithms. Bearing fault monitoring and diagnosis can then be realized. Currently, there are several signal-processing methods that are widely used: wavelet transform [4,5], stochastic resonance [6,7], and empirical mode decomposition (EMD) [8,9]. However, in wavelet transform, the choice of basic function and potential function parameters in stochastic resonance has great influence on signal analysis. Therefore, it cannot be defined as an adaptive signal-processing method. Empirical mode decomposition can be based on the signal itself. However, its modal-aliasing phenomenon seriously affects its application in actual signals.

Intrinsic time-scale decomposition (ITD) is an adaptive nonstationary nonlinear signal-processing method. Time–frequency analysis based on the intrinsic time-scale decomposition can quantitatively describe the relationship between frequency and time, accurately analyzing time-varying signals [10]. On the basis of these advantages, scholars introduced this method from the medical field to the fault diagnosis of mechanical signals [11,12,13,14,15,16,17,18,19,20,21,22]. For example, Lin and Chang published a rolling-bearing fault diagnosis method based on an enhanced kurtosis spectrum and intrinsic time-scale decomposition [11]. Duan and Yao et al. proposed a comprehensive eigentime decomposition method for the fault diagnosis of a gearbox under variable operating conditions [12]. Xiang and Qu et al. proposed intrinsic time-scale decomposition and singular-value decomposition for variable-condition gear fault diagnosis [13]. Zhang and Liu et al. proposed a complete diesel-engine fault-diagnosis method integrated with intrinsic time-scale decomposition [14]. Hu and Xiang et al. proposed ensemble intrinsic time-scale decomposition to the fault diagnosis of fan gear [15]. Tong, Cao, et al. proposed improved intrinsic time-scale decomposition combined with complex tree wavelet packet transform and singular-value decomposition and used it to diagnose rolling-bearing faults [16]. Liu and Zhang et al. proposed the use of intrinsic time-scale decomposition for diesel-engine fault diagnosis [17]. Bi and Ma et al. proposed to use complete ensemble intrinsic time-scale decomposition and detect gasoline-engine knock [18]. Yu and Liu proposed using sparse coding on the basis of intrinsic time-scale decomposition to diagnose weak bearing faults [19]. Yuan and Peng proposed the use of smooth intrinsic time-scale decomposition for the fault diagnosis of rolling bearings [20]. Lei and Zhou et al. used intrinsic time-scale decomposition to monitor tool wear during milling [21]. Ma et al. proposed complete ensemble intrinsic time-scale decomposition with adaptive noise (CEITDAN) that was applied to the feature extraction of rolling bearings [22].

However, there are currently still many problems in the study of intrinsic time-scale decomposition. Existing research results improved the decomposition of the intrinsic time scale. However, problems such as curve distortion and modal aliasing still need to be further improved. Therefore, in order to further allow for the better application of eigentime-scale decomposition algorithms to the early fault diagnosis of bearings, this paper proposes an intrinsic time-scale decomposition method based on time-varying filtering (TVF-ITD). It is applied to the framework of CEITDAN, improving the noise-reduction effect of intrinsic time-scale decomposition on strong background noise signals in order to improve the effectiveness of early fault diagnosis. And named it as complete ensemble intrinsic time-scale with adaptive white noise based on time-varying filtering (TVF-CEITDAN).

In the process of engineering practice, when the bearing has an early failure, the collected signal noise is relatively strong. The characteristic frequency of each type of fault is also different [23,24,25]. Therefore, adaptive analysis is also needed to extract different types of faults. In this paper, an TVF-CEITDAN method was used to decompose a signal. The use of adaptive white noise can offset noise in the collected signal. The collected signal contained both white and colored noise. Therefore, this method still retains some colored-noise components that are difficult to remove.

In order to further reduce the interference of noise on feature extraction, this paper proposes to introduce entropy calculation into the process of signal decomposition and reconstruction. Entropy is used as an analytical method to estimate the complexity of time series. It is widely used in the fault diagnosis of bearings [26,27,28,29,30]. At present, research on entropy is ongoing. The emergence of multiscale entropy in recent years provides a direction for its more accurate signal analysis [31,32,33,34,35]. Multiscale entropy can accurately measure the complexity of different mode components. It has high noise immunity and high consistency. At the same time, it is not restricted by large amounts of data. In the case of fewer data, a stabler entropy value can be obtained. Therefore, generalized refined composite multiscale sample entropy was chosen as the method of noise elimination after decomposition (GRCMSE) [36]. The entropy value of the generalized refined composite multiscale sample was calculated under 10,000 sets of white noise as the threshold, used in the decomposition of subsequent signals. This can further filter out residual noise in a decomposed signal through the verification of analog and measured vibration signals. The proposed method in this paper can accurately extract early fault features under a strongly noisy background. Compared with other methods, it shows better practicability.

The rest of this article is organized as follows. Section 2 introduces the principles of the ITD algorithm, GRCMSE, and the group-optimization algorithm. Section 3 introduces the proposed method. In Section 4, the effectiveness of the proposed method is verified by analog and measured signals. Lastly, the conclusions are drawn in Section 5.

## 2. Related Work

### 2.1. Intrinsic Time Scale Decomposition

ITD is adaptive to nonstationary signals. The method decomposes the vibration signal into a series of proper rotation components (PRCs) with physical significance and the sum of monotonous trend residuals. After the decomposition result is obtained, spectrum analysis can be performed on any PRC in order to obtain amplitude and frequency-modulation characteristics that are difficult for the original signal to show [37].

Suppose the noise signal to be processed is $X_{t}$, which is a set of discrete data composed of real numbers. All extreme points in $X_{t}$ are found, corresponding signal moments are $\tau_{k}$, (k=1,2,…,M), and M are the total number of signal extremes. First, L is defined as the baseline extraction operator; then, let $\tau_{0}$ be the first intrinsic scale of signal $X_{t}$ and be decomposed as:

(1) $$X_{t}=LX_{t}+\left(1-L\right)X_{t}=L_{t}+H_{t},$$

where $L_{t}=LX_{t}$ and $H_{t}=\left(1-L\right)X_{t}$ are the baseline extraction signal and the PRC, respectively. In the first decomposition, a baseline extraction signal is removed from original detection signal $X_{t}$ to obtain the PRC. Using $X_{k}$ and $L_{k}$ is equivalent to $X\left(\tau_{k}\right)$ and $L\left(\tau_{k}\right)$. To make $X_{t}$ meaningful in $t\in \left[0,\tau_{k+2}\right]$, both $L_{t}$ and $H_{t}$ are defined to be on $\left[0,\tau_{k}\right]$. On interval $\left[\tau_{k},\tau_{k+1}\right]$ of continuous extreme points, we define the piecewise linear baseline extraction operator as follows:

(2) $$LX_{t}=L_{t}=L_{k}+\frac{L_{k+1}-L_{k}}{X_{k+1}-X_{k}}\left(X_{t}-X_{k}\right)$$

Among them:

(3) $$L_{k+1}=\sigma \left[X_{k}+\left(\frac{\tau_{k+1}-\tau_{k}}{\tau_{k+2}-\tau_{k}}\right)\left(X_{k+2}-X_{k}\right)\right]+\left(1-\sigma \right)X_{k+1},$$

where σ is the linear scaling used to control the amplitude of the PRC; σ∈[0,1] usually takes the value 0.5. The decomposed PRC represents the local relatively high frequency component in the original detection signal, that is, the PRC. The baseline signal is used as the next original signal to continue the decomposition. Obtain a series of PRCs arranged in different frequency ranges from high to low. When iterating to produce a monotonous residual trend signal, decomposition ends. The decomposition process of the entire intrinsic time scale of signal $X_{t}$ can be as follows:

(4) $$\begin{array}{l} LX_{t}=LX_{t}+HX_{t}=\left(H+L\right)LX_{t}+HX_{t}\\ =\left[H\left(1+L\right)+L^{2}\right]X_{t}=\left(H\sum_{k=0}^{p-1}L^{k}+L^{p}\right)X_{t}\\ =H_{t}^{1}+H_{t}^{2}+H_{t}^{3}+H_{t}^{4}+\ldots +H_{t}^{p}+L_{t}^{p} \end{array}$$

where $H_{t}^{k}$ is the k-th PRC, $L_{t}^{p}$ is the residual component, and the number of PRCs after the decomposition is p.

### 2.2. Generalized Refined Composite Multiscale Sample Entropy

Multiscale sample entropy (MSE) can measure the complexity of time series and effectively detect small changes. When a rolling bearing fails, the complexity of nonlinear dynamics also changes [38]. Therefore, MSE is very suitable for the feature extraction of rolling-bearing faults. Experiment results showed that MSE can overcome the shortcomings of single-scale analysis of sample entropy, and analytical results are more accurate. However, applying MSE to the feature-extraction process of rolling bearings still has the two following defects: (1) Through the coarse-graining process of homogenized data, the dynamic mutation behavior of the original signal is “neutralized” to a certain extent, making the estimated entropy value biased. (2) The stability of MSE increases with an increase in coarse-grained scale factor. In order to overcome this shortcoming, the second moment (variance) is used in the coarse-grained step. Instead of the first moment (average value), the generalized MSE (GMSE) algorithm is proposed [39], which still has some shortcomings. GMSE depends heavily on the length of the time series, and the GMSE value may be uncertain or unreliable. The probability of invalid entropy may increase. In order to resolve these shortcomings, this paper applied the GRCMSE algorithm [36].

The process of the multiscale sample entropy algorithm is as follows:

(1) For time series {x(i),i=1,2,⋯,N}, following formula is used to define coarse-grained series $y^{\left(s\right)}$:
  (5) $$y_{j}^{\left(s\right)}=\frac{1}{s}\sum_{i=\left(j-1\right)s+1}^{js}x_{i},1\leq j\leq \frac{N}{s}$$
  where s is the scale factor.
(2) The sample entropy of coarse-grained sequence $y^{\left(s\right)}$ is calculated under different scale factors s, that is, multiscale sample entropy:
  (6) $$E_{MSE}\left(x,s,m,r\right)=E_{SE}\left(y^{\left(s\right)},m,r\right)=-\ln\left(n_{s}^{\left(m+1\right)}/n_{s}^{\left(m\right)}\right),$$
  where m is the embedding dimension; r is the similarity tolerance; $E_{SE}\left(\cdot \right)$ is the sample entropy value; $n_{s}^{\left(m\right)}$ and $n_{s}^{\left(m+1\right)}$ are the numbers of m- and m+1-dimensional space vectors of the coarse-grained sequence, respectively.

Generalized multiscale sample entropy (GMSE) is the calculation of the mean value of the coarse-grained process of multiscale sample entropy, which is extended to the second moment in order to overcome the shortcomings of “neutralizing” the dynamic mutation behavior of the original signal caused by the coarse-grained method of the homogenized data. The specific calculation steps are as follows:

(1) For time series {x(i),i=1,2,⋯,N}, the following formula is used to calculate generalized coarse-grained series $y_{G}^{\left(s\right)}$:
  (7) $$\begin{array}{l} y_{G}^{\left(s\right)}\left(j\right)=\frac{1}{s}{\sum_{i=\left(j-1\right)s+1}^{js}\left(x_{i}-{\bar{x}}_{i}\right)}^{2}\\ 1\leq j\leq \frac{N}{s},s\geq 2,{\bar{x}}_{i}=\frac{1}{s}\sum_{h=0}^{s-1}x_{i+h} \end{array}$$
(2) The sample entropy of generalized coarse-grained sequence $y_{G}^{\left(s\right)}$ is calculated under different scale factors s, that is, generalized multiscale sample entropy:
  (8) $$E_{GMSE}\left(x,s,m,r\right)=E_{SE}\left(y_{G}^{\left(s\right)},m,r\right)=-\ln\left(n_{G,s}^{\left(m+1\right)}/n_{G,s}^{\left(m\right)}\right).$$

This aims at the second deficiency of the multiscale sample entropy algorithm. On the basis of GMSE, considering multiple coarse-grained sequences under a unified scale factor, the obtained entropy value is stabler and more accurate; for scale factor s, all m- and m+1-dimensional generalized sequences are first averaged under the scale factor, the $n_{G,h,s}^{\left(m\right)}$ and $n_{G,h,s}^{\left(m+1\right)}$ of coarse-grained sequence $y_{G,h}^{\left(s\right)}$. Then, entropy is calculated to reduce the probability of invalid entropy. The above analysis is GRCMSE, and the specific steps are as follows:

(1) For time series {x(i),i=1,2,⋯,N}, the following formula is used to calculate generalized composite coarse-grained $y_{G,h}^{\left(s\right)}=\left\{y_{G,h,j_{1}}^{\left(s\right)},y_{G,h,j_{2}}^{\left(s\right)},\cdots ,y_{G,h,j_{s}}^{\left(s\right)}\right\}$:
  (9) $$\begin{array}{l} y_{G,h,j}^{\left(s\right)}\left(j\right)=\frac{1}{s}\sum_{i=\left(j-1\right)s+h}^{js+h-1}{\left(x_{i}-{\bar{x}}_{i}\right)}^{2}\\ 1\leq j\leq \frac{N}{s},2\leq h\leq s,{\bar{x}}_{i}=\frac{1}{s}\sum_{h=0}^{s-1}x_{i+h} \end{array}$$
(2) In the range of 1≤h≤s, the average values ${\bar{n}}_{G,h,s}^{\left(m\right)}$ and ${\bar{n}}_{G,h,s}^{\left(m+1\right)}$ of $n_{G,h,s}^{\left(m\right)}$ and $n_{G,h,s}^{\left(m+1\right)}$ are calculated to obtain the GRCMSE value of time series x(i) under scale factor *s*:
  (10) $$\begin{array}{l} E_{GRCM}=-\ln\left({\bar{n}}_{G,h,s}^{\left(m+1\right)}/{\bar{n}}_{G,h,s}^{\left(m\right)}\right)\\ {\bar{n}}_{G,h,s}^{\left(m\right)}=\frac{1}{s}\sum_{h=1}^{s}n_{G,h,s}^{\left(m\right)},{\bar{n}}_{G,h,s}^{\left(m+1\right)}=\frac{1}{s}\sum_{h=1}^{s}n_{G,h,s}^{\left(m+1\right)} \end{array}$$

### 2.3. Coyote Optimization Algorithm

The coyote optimization algorithm (COA) is a new group optimization algorithm proposed in 2018 by Pierezan et al., inspired by the behavior of coyotes in North America [40]. The algorithm simulates existing coyote populations and their evolution, including heuristic random coyote-population grouping, growth, birth, and death, original-group driving-away, and new-group acceptance behavior. The COA focuses on the social structure and cultural exchange of coyotes and does not search according to social hierarchy and ruling rules. After the coyotes are equally divided into several subgroups, the alpha animal and cultural trends of the subgroups are independently determined. Two different random wolves in the group form cultural differences with the alpha coyote and the cultural trend, respectively. As a disturbance catalyzes the growth of a coyote, two different coyotes in the group are randomly selected to give birth to cubs under the influence of a specific environment, and the birth and death of coyotes are synchronized. Whether the cub survives depends on whether its adaptability is better than that of any coyote in the group. Either the cub dies, or the oldest coyote in the group dies. Individual coyotes are driven out of the group to enter other groups, which plays a role in experience exchange. Through the cyclic evolution of growth, birth, and death, the exclusion of the original group and the acceptance of the new group obtains the coyote with the best fitness as a solution to the optimization problem. In COA, decision variables are represented by coyote social-state factors in each dimension of the solution vector. Each coyote represents a candidate solution to the problem. The advantages and disadvantages of the candidate solutions depend on the coyotes’ social adaptability.

COA groups the initialized coyote populations according to the principle of random equal distribution. So, after setting the number of coyotes in group $N_{p}\in N^{*}$ and the number of coyotes in a single group $N_{c}\in N^{*}$, we can obtain $N_{p}\times N_{c}$ individual coyotes. The initial social conditions of these individual coyotes are randomly set. This is achieved by assigning random values in the search space to various social-state factors of the coyotes. Equation (11) expresses the assignment method of the j-th dimension of the c coyote in the p package:

(11) $$soc_{c,j}^{p}=lb_{j}+r\cdot \left(ub_{j}-lb_{j}\right),$$

where $ub_{j}$ and $lb_{j}$ represent the upper and lower bounds, respectively, of the j-th dimension of the decision variable, and r is uniformly distributed in [0,1]. On this basis, the social adaptability of coyotes is evaluated according to Equation (12):

(12) $$fit_{c}^{p}=f\left(soc_{c}^{p}\right)$$

The growth of the coyotes in the group is the result of cultural interaction. It is affected by the alpha wolf, the cultural trend $\left(cult^{p,t}\right)$ of this group, and two different coyotes ($cr_{1}$ and $cr_{2}$) randomly in the group. The cultural difference between alpha wolf and random wolf $cr_{1}$ forms impact factor $\delta_{1}$, and the cultural difference between $cult^{p,t}$ and random wolf $cr_{2}$ forms impact factor $\delta_{2}$, namely:

(13) $$\delta_{1}=alpha^{p,t}-soc_{cr1}^{p,t}$$

(14) $$\delta_{2}=cult^{p,t}-soc_{cr2}^{p,t}$$

The alpha wolf is the coyote with the best environmental adaptation in the group. When solving the minimization problem, it is defined as:

(15) $$alpha^{p,t}=\left\{soc_{c}^{p,t}|\arg_{c=\left\{1,2,\ldots ,N_{c}\right\}}\min f\left(soc_{c}^{p,t}\right)\right\}.$$

Cultural trends provide conditions for coyotes in the group to share information, composed of the median values of the social conditions of all coyotes in the group, and it is the performance of algorithmic swarm intelligence. The specific calculation formula is:

(16) $$cult_{j}^{p,t}=\{\begin{array}{c} O_{\frac{\left(N_{c}+1\right)}{2}}^{p,t},\begin{array}{ccc} N_{c} & is & odd \end{array}\\ \frac{O_{\frac{\left(N_{c}\right)}{2},j}^{p,t}+O_{\frac{\left(N_{c}+1\right)}{2},j}^{p,t}}{2}\begin{array}{cc} , & otherwise \end{array} \end{array},$$

where $O^{p,t}$ represents the social condition under which [1,D] in the p-th package in the t-th iteration is sorted by dimension.

Therefore, the social state of a coyote after growing up is shown in Equation (17):

(17) $$new\_soc_{c}^{p,t}=soc_{c}^{p,t}+r_{1}\cdot \delta_{1}+r_{2}\cdot \delta_{2}$$

where $r_{1}$ and $r_{2}$ are the weights of the influence of alpha wolf and the cultural trend within the group, respectively; they are random numbers that obey [0,1], uniformly distributed.

COA still uses a greedy algorithm to determine whether the growth of a coyote is allowed. Equation (18) evaluates the growth state of a coyote. In Equation (19), coyotes with better environmental adaptability are retained to participate in the subsequent processes of growth, birth, and death, exclusion from the original group, and acceptance into the new group:

(18) $$new\_fit_{c}^{p,t}=f\left(new\_soc_{c}^{p,t}\right)$$

(19) $$soc_{c}^{p,t+1}=\{\begin{array}{c} new\_soc_{c}^{p,t}\begin{array}{cc} , & new\_fit_{c}^{p,t}<fit_{c}^{p,t} \end{array}\\ soc_{c}^{p,t}\begin{array}{cc} , & otherwise \end{array} \end{array}$$

Following the laws of nature, coyotes in the group give birth to cubs when they grow up, and they also face death. Two parent coyotes selected at random in the group give birth to cubs in a specific environment. The specific birth method of the cubs is:

(20) $$pup_{j}^{p,t}\{\begin{array}{c} soc_{r_{1},j}^{p,t}\begin{array}{cccc} , & rand_{j}<P_{s} & or & j=j_{1} \end{array}\\ soc_{r_{2},j}^{p,t}\begin{array}{cccc} , & rand_{j}\geq P_{s}+P_{a} & or & j=j_{2} \end{array}\\ R_{j}\begin{array}{cc} , & otherwise \end{array} \end{array},$$

where $r_{1}$ and $r_{2}$ are two random coyotes in the current group; $j_{1}$ and $j_{2}$ represent two random dimensions; $rand_{j}$ is a random number uniformly distributed in [0,1]; $R_{j}$ is a random number within the bounds of the j-th dimensional decision variable, representing the impact of the reproductive environment on the cubs; and $P_{s}$ and $P_{a}$ are scattering probability and correlation probability, respectively, which determine the degree of cultural diversity of coyotes in the group:

(21) $$P_{s}=\frac{1}{D},P_{a}=\frac{1-P_{s}}{2}$$

COA synchronizes the birth and death of coyotes to maintain a stable population size. According to the standard design of Algorithm 1, older coyotes have a higher mortality rate. Among them, ω and φ represent the set of coyotes whose environmental adaptability is not as good as that of the cubs and the number of coyotes in the set, respectively. If there are two or more coyotes of similar age in ω, the coyote with the worst adaptability is still set to die according to the greedy algorithm.

**Algorithm1.** Birth and Death of Coyotes.
1:Compute *ω* and *φ*2:*ifφ* = 1 *then*3:The pup survives and the only coyote in *ω* dies.4:*else ifφ* > 1 *then*5:The pup survives and the oldest coyote in *ω* dies.6:
*else*
7:The pup dies.8:
*end if*



The entire population is unstable in which individual coyotes are driven out of the group and accepted into the new group. The more coyotes there are in the group, the higher the probability $P_{e}$ of this happening is:

(22) $$P_{e}=0.005\cdot N_{c}^{2}$$

Different from sharing cultural information within groups, this mechanism can promote the global cultural exchange of coyote population. In order to ensure that A is between 0 and 1, the number of coyotes in each group is required to be up to 14 [41]. The COA process uses pseudocode as shown in Algorithm 2:

**Algorithm2.** COA Pseudo Code.
1:Define control parameters *N_p_*, *N_c_*, and maximal iterations *N_Max*2:Initialize *N_p_* packs with *N_c_* coyotes each and verify coyote adaptation3:While *t* < *N_Max*, do4:*forp* = 1:*N_p_*5:determine *alpha* and culture tendency6:*forc* = 1:*N_c_*7:Update the social condition8:Evaluate the new social condition9:Adaptation10:
*end for*
11:Generate the pup considering intrinsic and extrinsic influence12:The pup dies or the oldest coyote dies13:
*end for*
14:Transition between packs15:Update coyote ages16:
*end while*
17:Select the best adapted coyote


To sum up, the COA structure is novel and can better balance global exploration and local development. The coyote growth model considers more factors and strengthens its ability to jump out of the local optimum. When giving birth to cubs, the influence of the external environment makes the algorithm capable of exploring well. An individual coyote is expelled and reaccepted by the new group, playing a role in overall cultural exchange, avoiding information convergence within the group and the optimization of the mantis oriole style. The local development of individual coyotes then has global awareness. Therefore, the algorithm has good value.

## 3. Proposed Method

This paper proposes an improved time-varying, filter-based self-adaptive white-noise, fully integrated eigentime-scale decomposition algorithm, and an improved coyote optimization algorithm. The two methods are organically combined with the generalized fine composite multiscale sample entropy. The filtering effect was improved to extract early fault characteristic frequencies of rolling bearings under strong noise. This section includes three parts. The first part is an improved, time-varying filter-based self-adaptive white-noise fully integrated intrinsic time-scale decomposition algorithm; the second part is a coyote optimization algorithm based on gradient-based optimizer (GBO)–sine–cosine optimization (SCA) optimization; the third part proposes the specific implementation of feature extraction.

### 3.1. Fully Integrated Intrinsic Time-Scale Algorithm with Adaptive White Noise Based on Time-Varying Filtering

Because the ITD method is the same as the EMD method, there are problems of mode components lacking physical meaning and mode aliasing. At the same time, ITD uses linear interpolation for curve fitting. As a result, the decomposed signal has serious curve distortion in low-frequency components. Therefore, this article was inspired by the TVF–EMD method [41]. TVF was applied to the ITD method in order to further improve the modal aliasing. This article applied the TVF–ITD method to the CEITDAN technical framework. The specific methods proposed in this article are as follows.

The intrinsic time-scale decomposition of time-varying filtering is essentially performed by constructing a low-pass filter of which the cut-off frequency changes with time to complete the iterative removal of linear interpolation in the ITD process and replace the inherent rotation component with the local narrowband signal as the iteration stop condition. For a given arbitrary multicomponent signal A, it can be expressed as a two-component signal:

(23) $$x\left(t\right)=A\left(t\right)e^{j\varphi \left(t\right)}=a_{1}\left(t\right)e^{j\varphi_{1}\left(t\right)}+a_{2}\left(t\right)e^{j\varphi_{2}\left(t\right)}$$

Therefore, only the decomposition process of the two-component signal needs to be considered. The basic steps of time-varying filtering eigentime-scale decomposition for two-component signals are as follows:

Step 1: Perform Hilbert transform on x(t) to obtain amplitude A(t) and phase φ(t) of the complex analytical signal. The derivative of the instantaneous phase obtains instantaneous frequency $\varphi^{\prime }\left(t\right)$.

(24) $$z\left(t\right)=A\left(t\right)e^{j\varphi \left(t\right)}=a_{1}\left(t\right)e^{j\varphi_{1}\left(t\right)}+a_{2}\left(t\right)e^{j\varphi_{2}\left(t\right)}$$

Step 2: Determine time $\left\{t_{\min}\right\}$, $\left\{t_{\max}\right\}$ and amplitude $A\left(\left\{t_{\min}\right\}\right)$, $A\left(\left\{t_{\max}\right\}\right)$ of the minimal and maximal values of amplitude curve A(t).

Step 3: Respectively interpolate the extreme points of A(t), and the resulting curves are $\beta_{1}\left(t\right)$ and $\beta_{2}\left(t\right)$, respectively.

Step 4: Calculate instantaneous mean value $a_{1}\left(t\right)$ and instantaneous envelope $a_{2}\left(t\right)$ according to equations (25) and (26).

(25) $$a_{1}\left(t\right)=\left[\beta_{1}\left(t\right)+\beta_{2}\left(t\right)\right]/2$$

(26) $$a_{2}\left(t\right)=\left[\beta_{2}\left(t\right)-\beta_{1}\left(t\right)\right]/2$$

Step 5:

(27) $$\eta_{1}\left(t\right)={\phi^{\prime }}_{1}\left(t\right)\left[a_{1}^{2}\left(t\right)-a_{1}\left(t\right)a_{2}\left(t\right)\right]+{\phi^{\prime }}_{2}\left(t\right)\left[a_{2}^{2}\left(t\right)-a_{1}\left(t\right)a_{2}\left(t\right)\right]$$

(28) $$\eta_{2}\left(t\right)={\phi^{\prime }}_{1}\left(t\right)\left[a_{1}^{2}\left(t\right)+a_{1}\left(t\right)a_{2}\left(t\right)\right]+{\phi^{\prime }}_{2}\left(t\right)\left[a_{2}^{2}\left(t\right)+a_{1}\left(t\right)a_{2}\left(t\right)\right]$$

Available local cutoff frequency ${\varphi^{\prime }}_{bis}\left(t\right)$:

(29) $${\phi^{\prime }}_{bis}\left(t\right)=\frac{{\phi^{\prime }}_{1}\left(t\right)+{\phi^{\prime }}_{2}\left(t\right)}{2}=\frac{\eta_{2}\left(t\right)-\eta_{1}\left(t\right)}{4a_{1}\left(t\right)a_{2}\left(t\right)}$$

The above steps are the construction of the time-varying filter.

Step 6: In order to eliminate mode aliasing caused by noise and other components, cutoff frequency ${\varphi^{\prime }}_{bis}\left(t\right)$ needs to be adjusted.

(1) Define time series $u_{i},i=1,2,3,\ldots$ of the maximal point of signal x(t);

If:

(30) $$\frac{\max\left({\phi^{\prime }}_{bis}\left(u_{i}:u_{i+1}\right)\right)-\min\left({\phi^{\prime }}_{bis}\left(u_{i}:u_{i+1}\right)\right)}{\left({\phi^{\prime }}_{bis}\left(u_{i}:u_{i+1}\right)\right)}>\rho$$

where ρ=0.25, $u_{i}$ is called a breakpoint. Let $e_{j}=u_{i}$, j=1,2,3,…, where $e_{j}$ denotes the sequence of discontinuities.

If ${\phi^{\prime }}_{bis}\left(u_{i+1}\right)-{\phi^{\prime }}_{bis}\left(u_{i}\right)>0$, then $e_{j}$ is the rising edge of ${\varphi^{\prime }}_{bis}\left(t\right)$; otherwise, $e_{j}$ is the falling edge of ${\varphi^{\prime }}_{bis}\left(t\right)$.

(2) If $e_{j}$ is the rising edge of ${\varphi^{\prime }}_{bis}\left(t\right)$, then ${\phi^{\prime }}_{bis}\left(e_{j-1}:e_{j}\right)$ is regarded as the lowest value; otherwise, $e_{j}$ is the falling edge of ${\varphi^{\prime }}_{bis}\left(t\right)$; then, ${\phi^{\prime }}_{bis}\left(e_{j}:e_{j+1}\right)$ is regarded as the lowest value. The rest of ${\varphi^{\prime }}_{bis}\left(t\right)$ is seen as a peak.

(3) Adjusted cut-off frequency ${\varphi^{\prime }}_{bis}\left(t\right)$ is obtained by interpolating between the peaks.

Step 7: reconstruct the signal according to adjusted cut-off frequency:

(31) $$h\left(t\right)=\cos\left\lfloor \int {\varphi^{\prime }}_{bis}\left(t\right)dt\right\rfloor .$$

Taking the extreme point of h(t) as the node, h(t) is divided into n segments, and the step size of each segment is m; n is called the order of B-spline function. Using Equations (32) and (33) for B-spline interpolation approximation, the approximation result is recorded as m(t), which represents the local mean curve:

(32) $$g_{m}^{n}\left(t\right)={\left[p_{m}^{n}*x\right]}_{↓m}*b_{m}^{n}\left(t\right)$$

(33) $$p_{m}^{n}={\left[{\left({\left[b_{n}^{m}*b_{m}^{n}\right]}_{↓m}\right)}^{-1}\right]}_{↑m}*b_{m}^{n}$$

(34) $$b_{m}^{n}\left(t\right)=\beta^{n}\left(t/m\right)$$

(35) $$b_{n}^{m}=\beta^{m}\left(t/n\right)$$

where ${\left[\cdot \right]}_{↓m}$ is downsampling, sampling every m point; ${\left[\cdot \right]}_{↑m}$ means oversampling, that is, m sampling points are inserted between every two sampling points.

Step 8: judge whether stop criterion θ(t)<ξ is satisfied, where ξ is the given bandwidth threshold. If satisfied, x(t) is PRC; if not, x(t)=x(t)−m(t) repeats Steps 1–7 until the stop criteria are met; x(t) satisfying the stopping criterion is PRC:

(36) $$\theta \left(t\right)=\frac{B_{Loughlin}}{\phi_{avg}\left(t\right)}$$

(37) $$\phi_{avg}\left(t\right)=\frac{a_{1}^{2}\left(t\right){\phi^{\prime }}_{1}\left(t\right)+a_{2}^{2}\left(t\right){\phi^{\prime }}_{2}\left(t\right)}{a_{1}^{2}\left(t\right)+a_{2}^{2}\left(t\right)}$$

(38) $$B_{Loughlin}=\sqrt{\frac{{a^{\prime }}_{1}^{2}\left(t\right)+{a^{\prime }}_{2}^{2}\left(t\right)}{a_{1}^{2}\left(t\right)+a_{2}^{2}\left(t\right)}+\frac{a_{1}^{2}\left(t\right)a_{2}^{2}\left(t\right){\left({\phi^{\prime }}_{1}\left(t\right)-{\phi^{\prime }}_{2}\left(t\right)\right)}^{2}}{{\left(a_{1}^{2}\left(t\right)+a_{2}^{2}\left(t\right)\right)}^{2}}}$$

**Figure 1:**
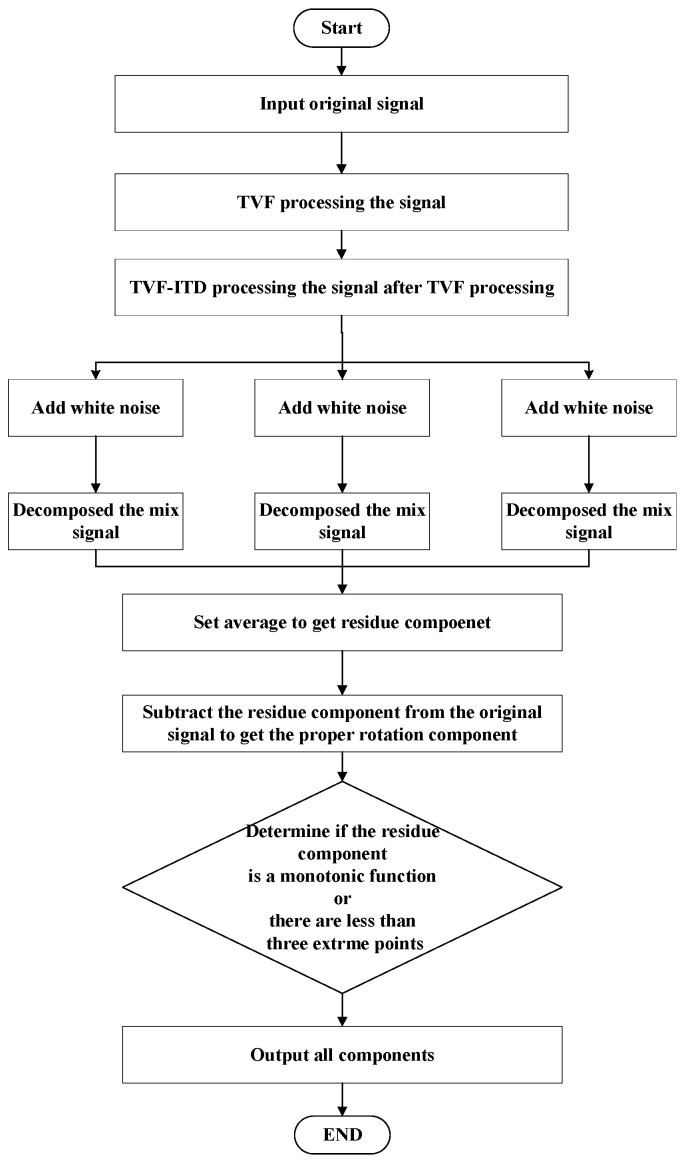
Flowchart of TVF-CEITDAN.

As mentioned earlier, the extraction and calculation of $L_{t}$ in the eigentime-scale algorithm were replaced by the TVF method proposed in this section. This can compensate for curve distortion and modal aliasing caused by monotonic linear interpolation. In order to further improve the modal aliasing effect, TVF–ITD is introduced into the CEITDAN ideological framework (TVF–CEITDAN). The specific flowchart is shown in Figure 1.

### 3.2. Coyote Optimization Algorithm Based on GBO–SCA Optimization

The proposed method in this paper is inspired by the GBO method proposed in [42] and introduced into the coyote optimization algorithm. The GBO method is an optimization algorithm that combines the gradient method and the population method and consists of two main parts. One of them is GSR based on the gradient search (GB) method. in the gradient search rule (GSR), the motion of the vector is controlled to better search and obtain a better position in the FEASIBLE region to improve the trend of exploration and accelerate the convergence of GBO. However, the rule was extracted from Newton’s gradient-based approach [43]. Based on the fact that many optimization problems are non-differentiable, the numerical gradient method is used instead of the direct function derivation method. However, the numerical gradient method uses one sample at a time for gradient descent. Therefore, to ensure accuracy, the stochastic random gradient descent method uses only one sample per training to determine the direction of the gradient. Local minima may be obtained, and the accuracy rate is not high. For convergence rate, the stochastic gradient descent method only iterates one sample at a time. Therefore, the iteration direction varies greatly and does not converge quickly to the local optimal solution. Therefore, in this paper, the GSR algorithm in the GBO method is proposed to be replaced by the positive cosine algorithm [44] to improve the overall convergence rate.

The sine–cosine optimization algorithm is a random optimization algorithm with a high degree of flexibility. The principle is simple, easy to implement, and can be easily applied to optimization problems in different fields. The optimization process of the sine–cosine optimization algorithm can be divided into two stages. In the exploration stage, the optimization algorithm quickly finds feasible regions in the search space by combining a random solution in all random solutions. During the development phase, the random solution gradually changes. The rate of change of the random solution is lower than the rate of the exploration phase. In the sine–cosine algorithm, the candidate solutions are first randomly initialized. Then, the value of the current solution in each dimension is updated according to the sine or cosine function, combined with random factors. The specific update equation is:

(39) $$X_{i}^{t+1}=\{\begin{array}{c} \begin{array}{cc} X_{i}^{t}+r_{1}\times \sin\left(r_{2}\right)\times \left|r_{3}P_{i}^{t}-X_{i}^{t}\right| & r_{4}<0.5 \end{array}\\ \begin{array}{cc} X_{i}^{t}+r_{1}\times \cos\left(r_{2}\right)\times \left|r_{3}P_{i}^{t}-X_{i}^{t}\right| & r_{4}>0.5 \end{array} \end{array},$$

where $X_{i}^{t}$ is the position of the current individual in the i-th dimension and t-th generation; $r_{2}$ is a random number from 0 to 2π; $r_{3}$ is a random number from 0 to 2; $r_{4}$ is a random number from 0 to 1; and $P_{i}^{t}$ means the position of the i-th dimension of the optimal individual position variable in the second iteration:

(40) $$r_{1}=a-t\frac{a}{T},$$

where a is a constant; t is the current iteration number; T is the maximal iteration number; and parameter $r_{1}$ indicates that the location area of the next solution is within or outside the current solution and the optimal solution. A smaller value of $r_{1}$ helps to enhance the local development capability of the algorithm. A larger $r_{1}$ value helps to improve the global exploration ability of the algorithm. At the same time, the $r_{1}$ value gradually decreases with the number of iterations, which balances the local development and global search capabilities of the algorithm. $r_{2}$, $r_{3}$ and $r_{4}$ are random factors, and $r_{2}$ defines how far the current solution is toward or away from the optimal solution. Parameter $r_{3}$ gives a random weight for the optimal solution to immediately emphasize the effect of $\left(r_{3}>1\right)$ or ignore the influence of the optimal solution of $\left(r_{3}<1\right)$ in defining the moving distance of the candidate solution. Parameter $r_{4}$ is an equal switch between sine and cosine functions.

For a given problem, the sine–cosine optimization algorithm randomly creates a series of candidate solutions. According to the sine and cosine functions, the value of each candidate solution in all dimensions is updated. The cyclic mode of sine and cosine functions allows for one solution to be repositioned around other solutions. This can ensure that the search is performed in the space between the two solutions, and it can converge to the global optimum faster. The algorithm flow is as follows:

Step 1: Population initialization. Suppose that the group size of the population is *m*, and *m* solutions are randomly generated in the range of [0,255]. Randomly set the initial position of each solution.

Step 2: Calculate the fitness of all solutions.

Step 3: The location of the solution is updated. Select the corresponding location update formula according to the $r_{4}$ value. Update the position of the candidate solution in each dimension. Recalculate the fitness values of all candidate solutions. In this way, the fitness of each solution and of the global optimal position is obtained.

Step 4: Compare and update the location of the global optimal solution. The fitness value of each updated solution is compared with that of the global optimal solution. If the fitness value of the current solution is greater than the global optimal fitness value, the position of the global optimal solution is updated.

Step 5: If the algorithm termination condition is satisfied, output the optimal solution; otherwise, repeat Steps 2–4.

Compared with the GSR algorithm in the GBO method, this method both improves convergence speed and reduces the parameters of the GBO method, improving the simplicity of calculation. We named this method GBO–SCA.

**Figure 2:**
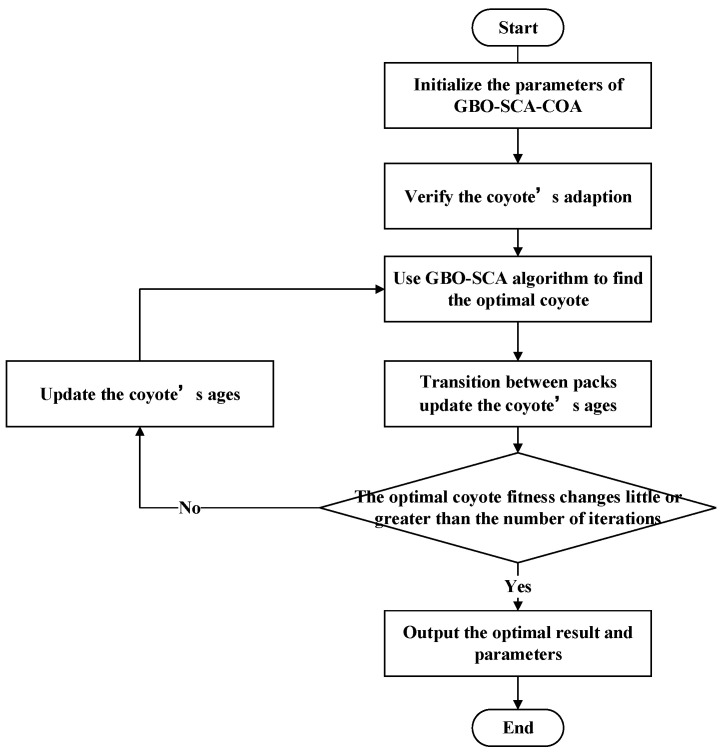
Flowchart of GBO-SCA-COA.

In summary, this article aims to improve the shortcomings of the COA method. By applying the GBO–SCA algorithm to the COA algorithm, the specific process is shown in Figure 2.

### 3.3. Specific Implementation Plan for Proposed Method

This paper proposes a new and improved method of eigentime-scale decomposition, and a new idea of applying generalized fine compound multiscale sample entropy to early fault diagnosis. Because the signal-to-noise ratio of each mode component in the CEITDAN ideological framework is fixed. Therefore, according to the threshold of the generalized fine composite multiscale sample entropy, it can be a fixed value when processing the agreement signal. There is no need to recalculate the threshold for each decomposition. According to the literature [36], scale factor s=25 is determined, and similarity tolerance is r=0.15×SD, where SD is the standard deviation. The method of early fault diagnosis of the overall rolling bearing in this article is as follows:

(1) Calculate the entropy value of the generalized fine composite multiscale sample of 10,000 groups of white noise, and the generalized fine composite multiscale sample entropy of the original signal. The average of the two is chosen as the threshold value.
(2) Use GBO–SCA–COA to adaptively optimize the TVF–CEITDAN parameters and decompose the original signal. The optimization criterion is to select the largest Si value for all components. The coyote chooses the smallest, so the negative of the largest weighted average of all time-domain parameters is chosen as the fitness value. The optimization process is to first set the search range of the TVF–ITD parameters, 0<ξ≤0.8, *n* = 5–30. Signal decomposition: calculate the Si of each mode component and save the minimal fitness value of each iteration. Determine whether the termination condition is satisfied; the termination condition is whether the current iteration number is greater than the termination iteration number. The minimal fitness and corresponding optimal parameters are extracted and substituted into TVF–CEITDAN. Re-decompose to obtain the final mode component.
(3) Calculate the entropy value of the generalized refined composite multiscale sample entropy for each signal component.
(4) Keep the mode components smaller than the threshold and reconstruct; then, perform the same operation as in the first step.
(5) Calculate the entropy value of the generalized fine composite multiscale sample of each mode component again and compare it with the threshold. If there are still mode components that are greater than the threshold, repeat from the first step until there is no pattern component greater than the threshold.
(6) Reconstruct the final decomposition result. Then, the reconstructed signal is transformed by the envelope spectrum to obtain the fault signal.

## 4. Results

### 4.1. Case A: Numerical Simulation

**Figure 3:**
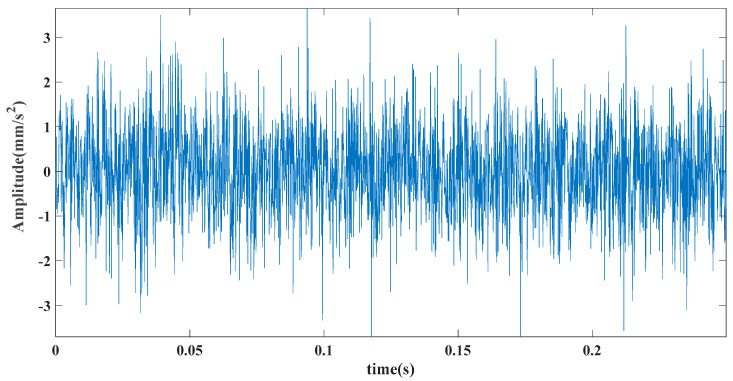
Time-domain diagram of analog signal.

**Figure 4:**
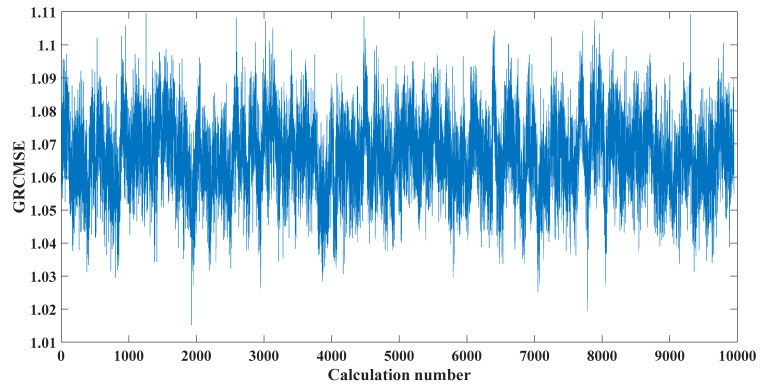
GRCMSE values of 10,000 groups of white-noise and analog signals.

**Figure 5:**
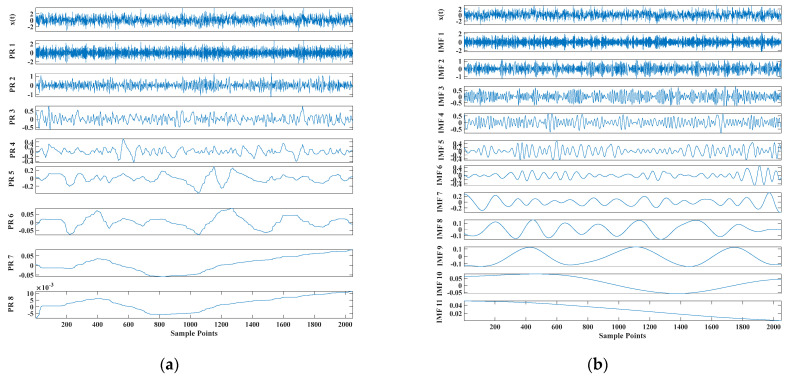

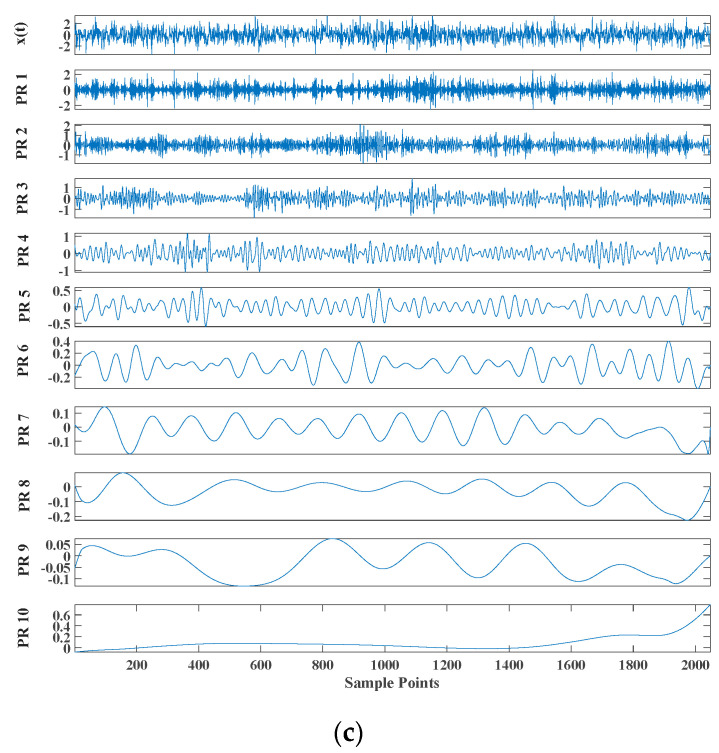
Decomposition results of (**a**) intrinsic time-scale decomposition (ITD), (**b**) CEEMDAN, and (**c**) TVF–CEITDAN.

In order to prove the superiority of the proposed algorithm in practical application, a bearing failure simulation signal was first selected, and complete ensemble empirical mode decomposition with adaptive noise (CEEMDAN), ITD, and TVF–CEITDAN decomposition, respectively, was used. The structure of the used bearing fault simulation signal is shown in Equation (43). In order to improve the objectivity of the three comparisons, orthogonality index (OI) and energy conservation index (ECI) were selected for the quantitative evaluation of the performance of different methods [45,46]. Theoretically speaking, after the algorithm decomposes the signal, the different obtained mode components should belong to a completely orthogonal relationship. That is, the orthogonality index is equal to zero. However, due to computer error and environmental interference, the orthogonality index cannot be zero [45]. Therefore, in practical application, the closer the orthogonal index is to 0, the more accurate the decomposition effect is; this shows that decomposition accuracy is not high. Therefore, from an energy point of view, the energy-preservation index can also evaluate the decomposition performance of different methods. Different from the orthogonal index, the value of the energy-conservation index is closer to 1. This shows that the less energy is lost in the decomposition process of this method, the more complete the decomposition result is. However, more than 1 indicates that there is a false component [46]. In summary, we used OI, ECI, and root mean square error (RMSE) to compare these methods. The OI and ECI are defined as:

(41) $$OI=Average\frac{\left|\langle {\mathrm{mod}}_{n}\left(t\right),{\mathrm{mod}}_{w}\left(t\right)\rangle \right|}{{\left\|{\mathrm{mod}}_{n}\left(t\right)\right\|}_{2}{\left\|{\mathrm{mod}}_{w}\left(t\right)\right\|}_{2}},n\neq w$$

(42) $$ECI=\frac{\sum_{t=0}^{T}\sum_{n=1}^{m}{\left|{\mathrm{mod}}_{n}\left(t\right)\right|}^{2}}{\sum_{t=0}^{T}{\left|x\left(t\right)-r_{m}\left(t\right)\right|}^{2}},$$

where x(t) represents the original signal, ${\mathrm{mod}}_{n}\left(t\right)$ represents the i-th mode component decomposed by decomposition algorithm, and $r_{m}\left(t\right)$ indicates residual component:

(43) $$\{\begin{array}{l} x\left(t\right)=\sum_{i=1}^{N}A_{i}s\left(t-iT-t_{i}\right)+n\left(t\right)\\ A_{i}=A_{0}\cos\left(2\pi f_{r}t+\varphi_{A}\right)+C_{A}\\ s\left(t\right)=e^{-2nf_{n}rt}\sin\left(2\pi f_{n}t+\varphi_{w}\right) \end{array},$$

where $A_{i}$ is the amplitude modulation with a period of $1/f_{r}$, the rotation frequency of the shaft is $f_{r}$; n(t) is random white noise; r is the damping coefficient of the system; T is the interval between consecutive shocks; $t_{i}$ is the rolling during the i-th period delay caused by body slip; $A_{0}$ and $C_{0}$ are arbitrary constants; and $f_{n}$ is the natural frequency of the system. Among them, *f_n_* = 3000 Hz, $f_{n}=3000$ Hz, $t_{i}=1$, $A_{0}=2$, $C_{0}=1$, r=0.005 fault characteristic frequency. The sampling rate was 8192 Hz, and the number of sample points was 2048, as shown in Figure 3. Figure 4 shows the GRCMSE values of 10,000 groups of white noise signals, where the minimum value is averaged with the GRCMSE value of the numerical signal as the threshold in this section. The threshold value used in this section is 0.43. Figure 5 shows the decomposition result of CEEMDAN, ITD, and TVF–CEITDAN.

**Figure 6:**
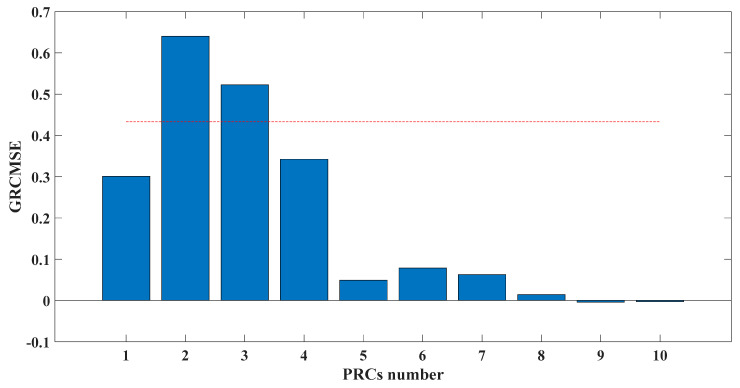
GRCMSE of TVF–CEITDAN decomposition result. (The red line in the graph indicates the threshold value).

**Figure 7:**
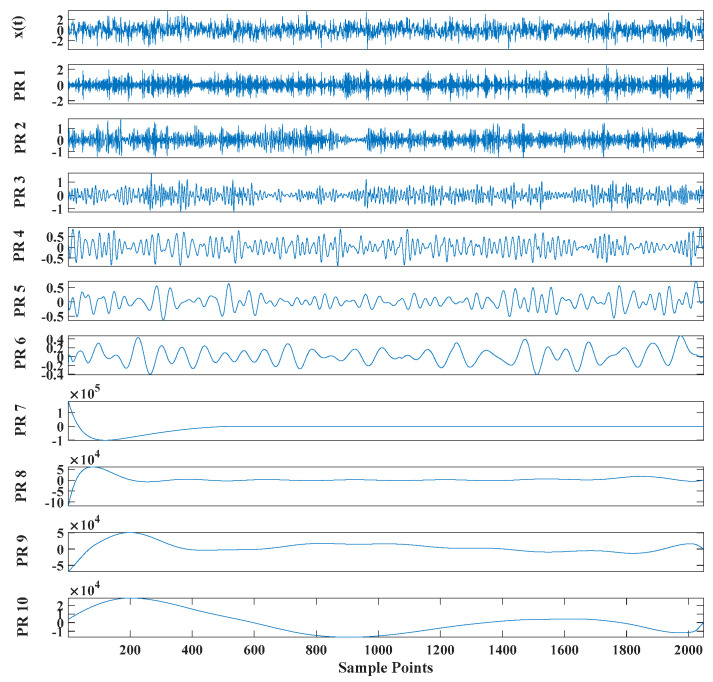
Reconstructed signal decomposed by TVF–CEITDAN.

**Figure 8:**
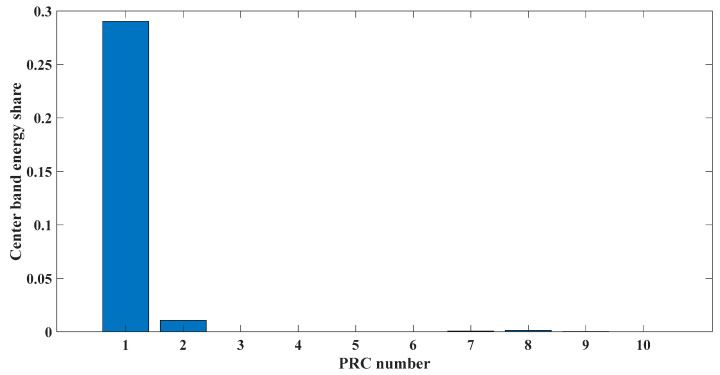
Energy ratio of central frequency band.

**Figure 9:**
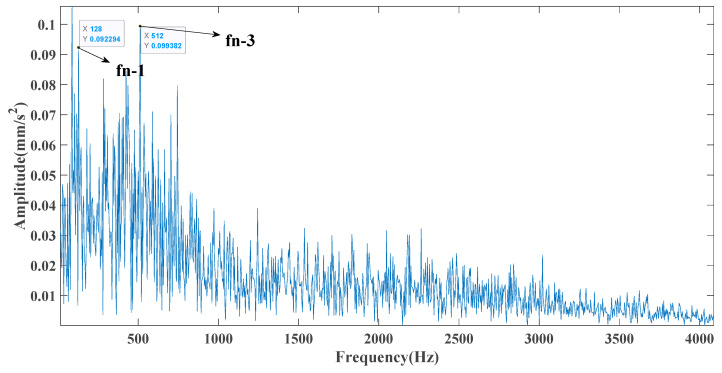
CEEMDAN envelope spectrum.

**Figure 10:**
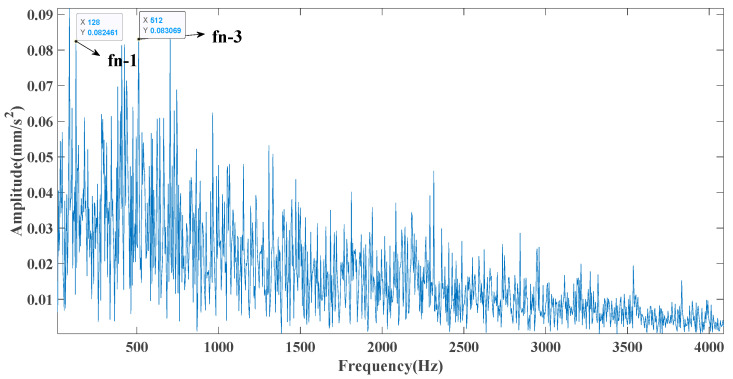
ITD envelope spectrum.

**Figure 11:**
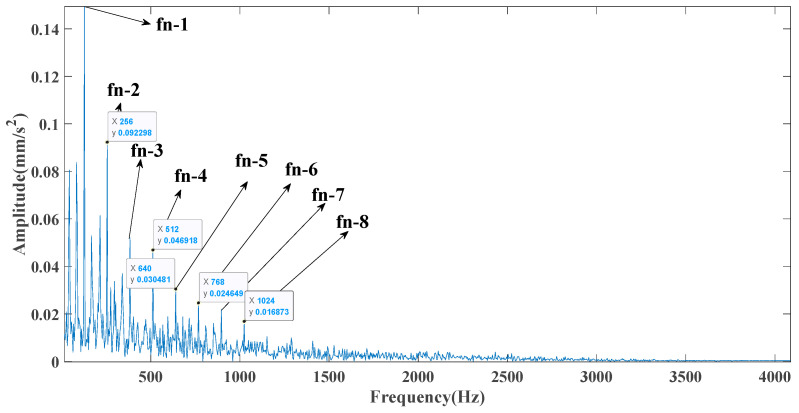
Envelope spectrum of proposed method.

**Figure 12:**
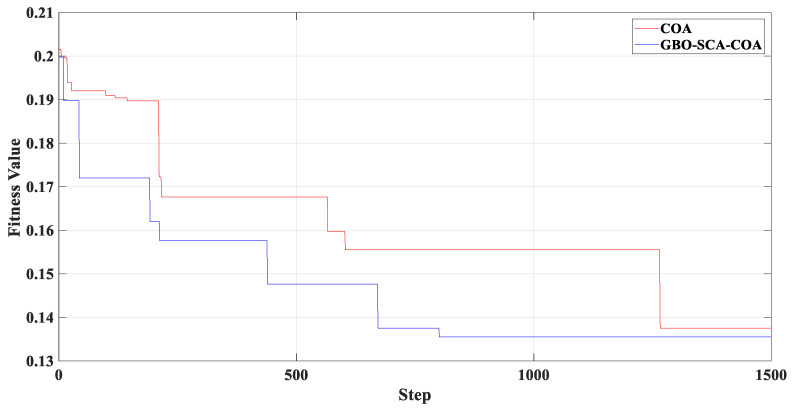
Fitness value of improved optimization algorithm and number of iterations.

Figure 5 shows that the curve decomposed by ITD caused low-frequency component distortion due to linear interpolation. CEEMDAN decomposed a total of 11 mode components, and the curve was smooth. However, IMF5 has modal aliasing, and the first three mode components did not highlight the impact signal. There was still strong noise interference. In the TVF–CEITDAN method, the first three components contained less noise, and the characteristics of the impact signal could be observed. According to the fault-diagnosis method proposed in this paper, the GRCMSE value of each rotation component of TVF–CEITDAN was calculated. Figure 6 shows that the GRCMSE values of PR2 and PR3 both exceeded the threshold. Therefore, the remaining rotation components were selected for signal reconstruction. TVF–CEITDAN was used to decompose again. The decomposition result is shown in Figure 7. In the application of the signal-decomposition algorithm, a key issue is how to select appropriate mode components to transform the envelope spectrum to extract the fault characteristic frequency and perform fault diagnosis. Therefore, this article used analysis of the central frequency band energy content contained in the different mode components to select the appropriate mode components for reconstruction, as shown in Figure 8. The central frequency band contained in PR1 accounted for the largest proportion of energy. Therefore, PR1 was selected to obtain the fault characteristic frequency for fault diagnosis. Figure 9, Figure 10 and Figure 11 are the envelope-spectrum results of CEEMDAN, ITD, and the proposed method. Figure 9 and Figure 10 show that, due to the strong noise of the analog signal, the result of the envelope spectrum obtained after decomposition could hardly show the characteristic frequency of the fault. There was no way to analyze and judge the type of failure. However, Figure 11 clearly shows the fault characteristic frequency and its multiplier. This also shows that the proposed method could maintain good effectiveness under strong noise interference, reducing it to ideal conditions. Figure 12 is a comparison diagram of the fitness value and the number of iterations of the GBO-SCA-COA in this paper. The proposed method could converge faster than traditional optimization algorithms can.

In order to objectively compare the practicability and effectiveness of the three methods, OI and RMI machine RMSE were used in this study, and results are shown in Table 1. As can be seen from Table 1, the ECI index of the TVF-CEITDAN method proposed in this paper reaches 0.9471, while the other two compared methods are only 0.5512 and 0.5915, indicating that the method proposed in this paper has the least energy loss in the signal decomposition process. For the OI index, the proposed method is much smaller than the remaining two methods and is closest to 1, which can be for the computational error of the computer, indicating good orthogonality and relative independence among the components. The RMSE of the proposed method in this paper is the smallest, which indicates that the decomposition error is the smallest. In the comparison of the output signal-to-noise ratio, the results of the proposed method in this paper are also the largest, which indicates that the noise reduction effect of the proposed method in this paper is better than the other two methods. The proposed method performed best in multiple indicators, and it can be further applied to actual engineering use.

### 4.2. Case B: Experimental Analysis

**Figure 13:**
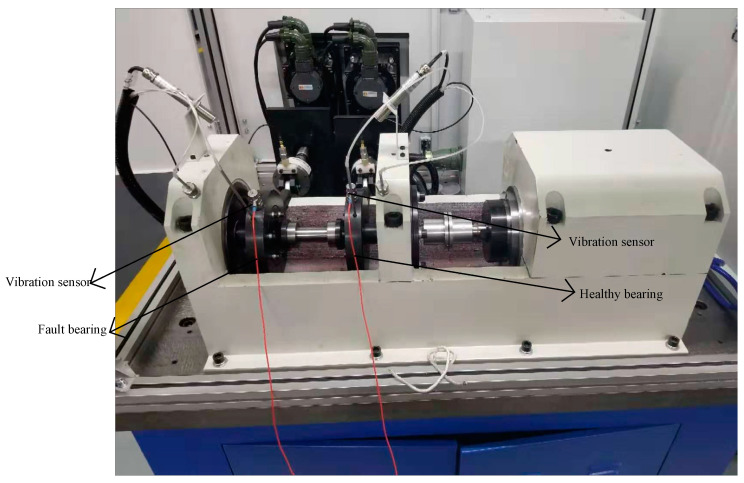
Experimental rig for real measurements.

In this section, we use an engineering test equipment for signal acquisition and fault diagnosis using a method consistent with the analog signal idea to demonstrate the effectiveness of the proposed method in this paper (Figure 13). The test platform consisted of a test bench and control system, which was mainly composed of the tested bearings, accompanying test bearings, test spindle, bearing outer ring fixture, drive unit, and loading system. During the test, there were four sets of bearings, divided into two sets of tested bearings and two sets of accompanying bearings. Two sets of loading systems respectively applied loads to the test bearings. The drive unit provided power for the whole test bench, and the loading system provided radial force for the test bearings, which rotated the spindle drive of the bearings and obtained the vibration signal of the bearings during the rotation process through vibration sensors. The drive unit provided bearing speed in the range of 1000–20,000 rpm, which was continuous. The loading system had a loading range of 0–6 KN, loading accuracy of ±2%, and it was continuously adjustable. The operating limit temperature of the test bench was 250 °C [47].

The vibration signal collected from the Fault bearing marked in Figure 13 is the signal used for the analysis in this paper, while the Healthy bearing is the accompanying bearing required for the test. Table 2 and Table 3 show the test-bearing parameters and characteristic frequency calculation formulas of rolling bearing, respectively.

**Figure 14:**
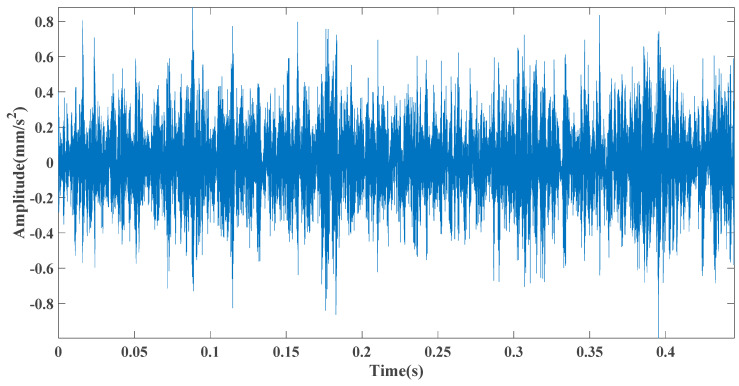
Time domain diagram of the test signal.

**Figure 15:**
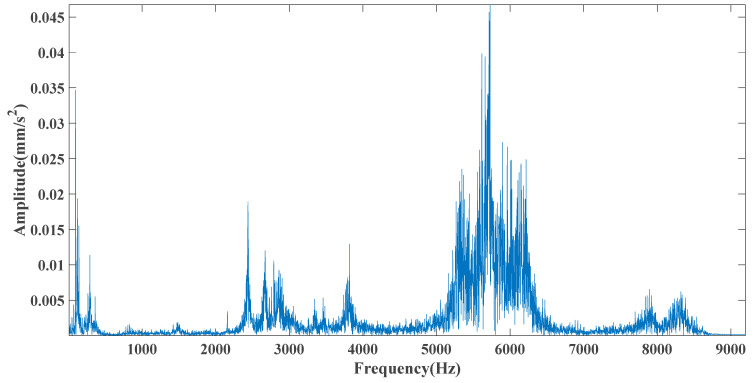
Frequency domain diagram of the test signal.

**Figure 16:**
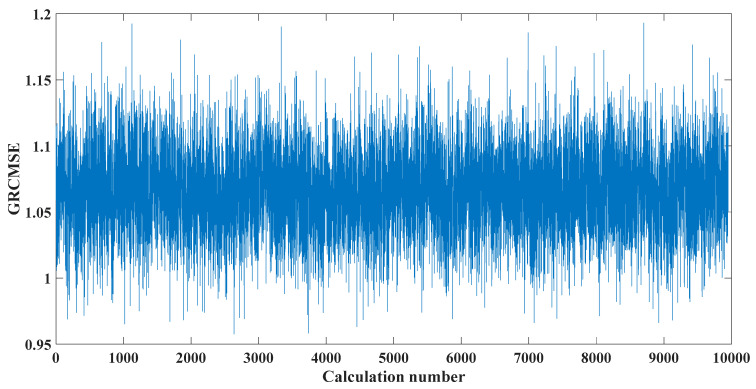
GRCMSE values of 10,000 groups of white noise and measured signals.

Sampling frequency was 18,400 Hz, data length was 8192 points, and bearing rotation speed was 3000 rpm. According to the parameters in Table 2 and the formula in Table 3, the bearing inner ring failure frequency can be calculated as 407 Hz. Due to computer error, the eigenfrequency in the envelope spectrum is actually 408Hz. Figure 14 and Figure 15 show the time domain and frequency domain plots of the acquired signals. From the above figure, it can be seen that the fault information is completely masked by the noise, and it is impossible to get the fault characteristic frequency visually and determine the fault type. Figure 16 shows the GRCMSE values of the 10,000 sets of white noise. The minimal value of the 10,000 sets of white noise and the GRCMSE value of the measured signal were selected, and their average value was used as the threshold value of 0.54.

**Figure 17:**
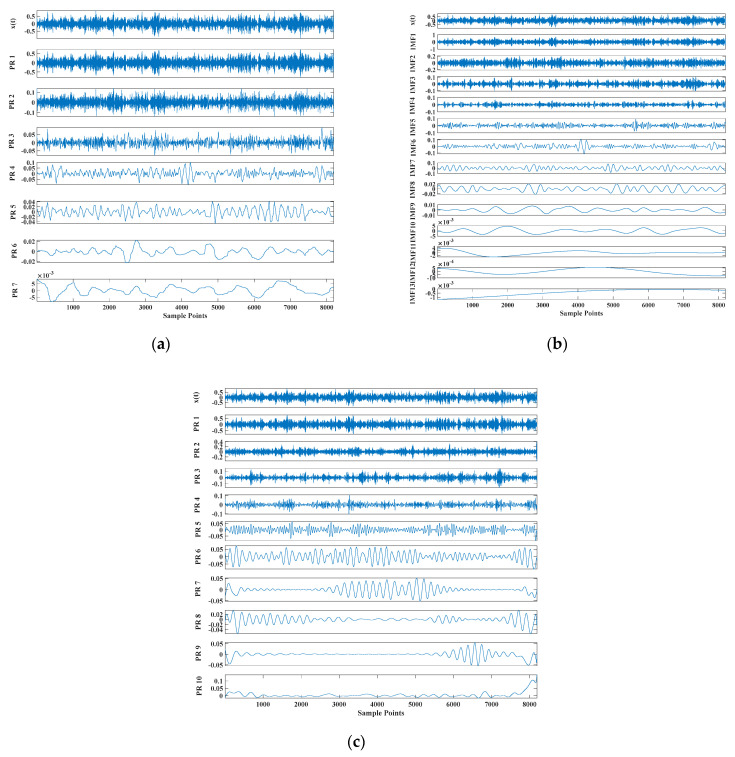
Decomposition results of (**a**) ITD, (**b**) CEEMDAN, and (**c**) TVF–CEITDAN.

**Figure 18:**
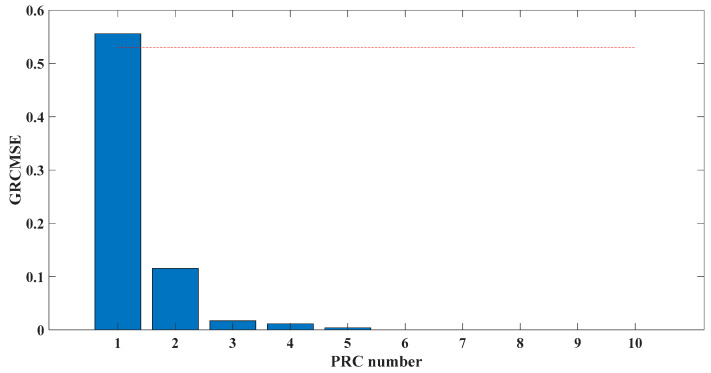
GRCMSE of each PRC. (The red line in the graph indicates the threshold value).

**Figure 19:**
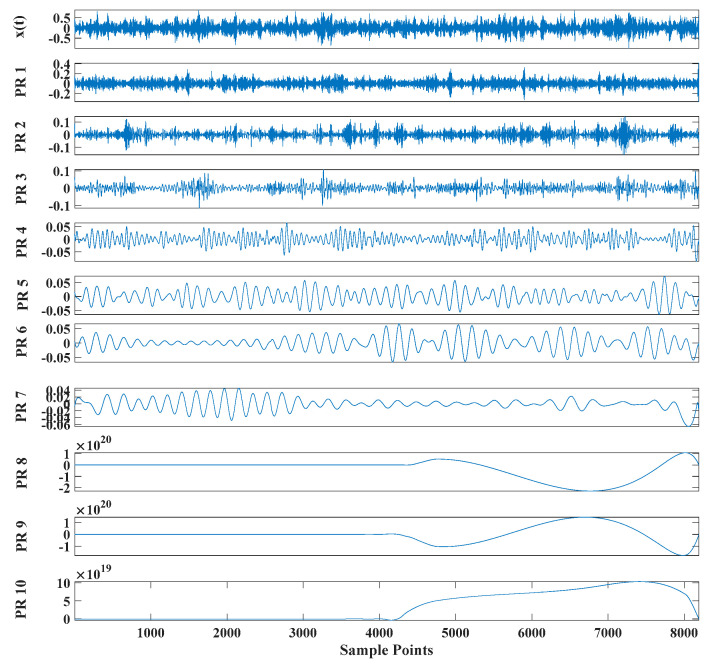
Reconstructed signal decomposed by TVF–CEITDAN.

**Figure 20:**
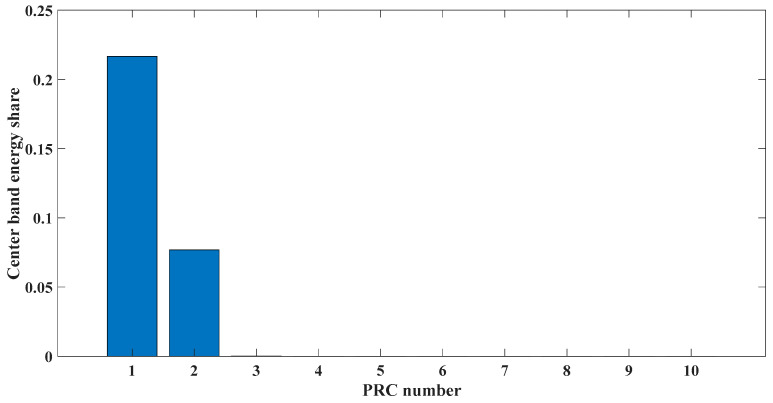
Energy ratio of central frequency band.

**Figure 21:**
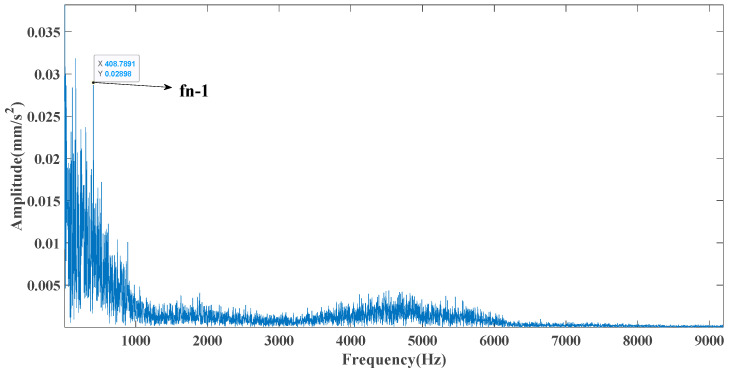
CEEMDAN envelope spectrum.

**Figure 22:**
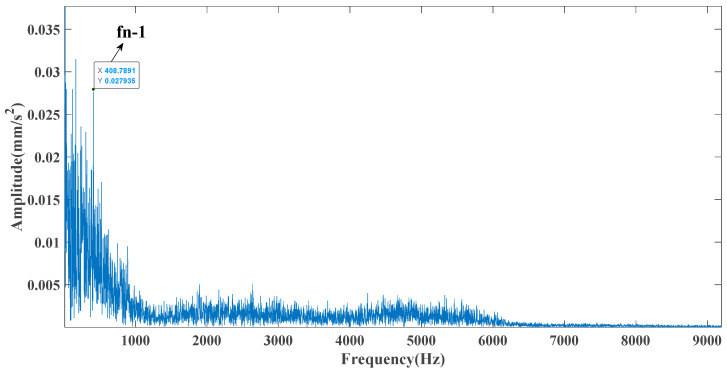
ITD envelope spectrum.

**Figure 23:**
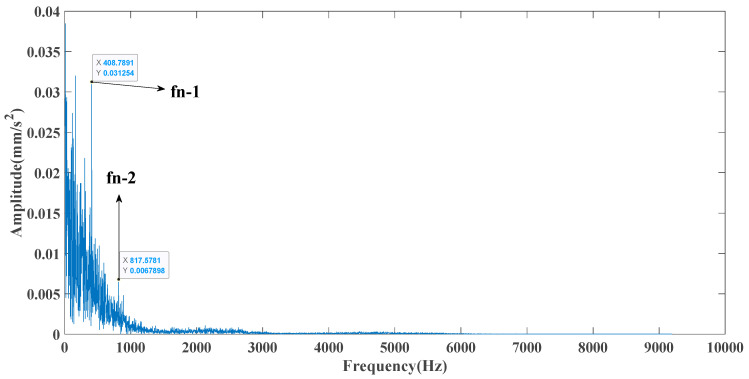
Envelope spectrum of proposed method.

**Figure 24:**
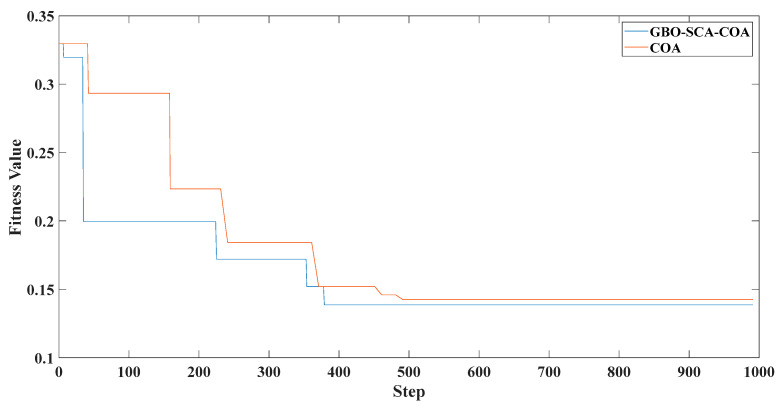
Fitness value of improved optimization algorithm and number of iterations.

Figure 17 shows that the decomposed curve by ITD caused low-frequency component distortion due to linear interpolation. CEEMDAN decomposed a total of 13 mode components. However, IMF4, IMF5, and IMF6 had modal aliasing. The first three mode components also had strong noise interference. In the TVF–CEITDAN method, the first three components contained less noise, and some of the characteristics of the impact signal could be observed. According to the proposed fault-diagnosis method, the GRCMSE value of each rotation component of TVF–CEITDAN was calculated as shown in Figure 18. The GRCMSE values of PR1 all exceeded the threshold. The remaining rotation components were then selected for signal reconstruction, and TVF–CEITDAN was used to decompose again. The decomposition result is shown in Figure 19. This part is the same as that in the previous section. The method of analyzing the energy ratio of the center frequency band contained in the different mode components was used to select the appropriate mode components for reconstruction, as shown in Figure 20. The central frequency band contained in PR1 accounted for the largest proportion of energy. Therefore, PR1 was selected for fault diagnosis. Figure 21, Figure 22 and Figure 23 are the envelope-spectrum results of CEEMDAN, ITD, and the proposed method. Figure 21 and Figure 22 show that, due to the stronger noise of the measured signal, the obtained envelope spectrum after decomposition could hardly show the fault characteristic frequency, and it was impossible to analyze and judge the fault type. CEEMDAN and ITD could only extract one characteristic frequency of the inner ring, and the gap between peak value and noise was small. However, Figure 23 clearly shows the fault characteristic frequency and its double frequency. This also shows that the proposed method maintained good effectiveness under strong noise interference, reducing noise interference to ideal conditions. Figure 24 is a comparison diagram of the fitness value and the number of iterations of the proposed improved coyote optimization algorithm. Our method could converge faster than traditional optimization algorithms can.

In order to objectively compare the practicability and effectiveness of the three methods, OI, ECI, and machine RMSE were used, and the results are shown in Table 4. As can be seen from Table 4, the proposed method in this paper performs consistently with the analog signal comparison results, and all the indexes perform best. The ECI index of the proposed method reaches 0.9389, while the other two comparison methods are only 0.5234 and 0.5625, indicating that the proposed method has the least energy loss in the process of signal decomposition, and because the measured signal is noisier and the signal-to-noise ratio is lower, the three methods are slightly lower in this index, which is also in line with the objective rule. For the OI index, the proposed method is much smaller than the remaining two methods and is closest to 1, which can be the calculation error of the computer, indicating good orthogonality and relative independence among the components. The RMSE of the method proposed in this paper is the smallest, which indicates that the decomposition error is the smallest. Since the input and output signal-to-noise ratios cannot be calculated for the measured signals, the output signal-to-noise ratio is not used as a comparison index to compare the three methods in this section. The proposed method performed best in multiple indicators and it can be further applied to actual engineering use.

## 5. Conclusions

This study proposed a new method for the early fault diagnosis of rolling bearings with the three following aspects: an improved eigentime-scale decomposition algorithm, an improved coyote optimization algorithm, and generalized fine composite multiscale sample entropy to analyze the noise-reduction effect. Through simulation signals and an engineering test platform, the effectiveness of the proposed method was fully proven. The proposed method takes generalized fine composite multiscale sample entropy as the objective function and removes the rotation component dominated by noise after decomposition. It carries out a second decomposition, fully retaining the weak signal of early bearing failure, and reduces the influence of noise. Through a comparison of different physical indicators, the purpose of the quantitative analysis of the method is obtained. The proposed method can be effectively applied to engineering practice. In future research, we will focus on the applicability of this method in the case of mixed failures. A set of feasible early failure and hybrid failure diagnosis methods for rolling bearings are proposed.

## Figures and Tables

**Table 1:** Index comparison table.

-	ECI	OI	RMSE	Output SNR
TVF–CEITDAN	0.9471	0.0054	0.0802	7.8293
CEEMDAN	0.5512	0.7666	0.3223	1.7135
ITD	0.5915	0.2876	0.2870	1.7978

**Table 2:** Test bearing parameters.

Ball Number N	Pitch Diameter D	Roller Diameter d	Contact Angle α
14	46	7.5	0

**Table 3:** Bearing-failure characteristic-frequency calculation formulas.

Bearing Fault	Equation
Bearing cage (FTF)	$\frac{1}{2}f_{r}\left[1-\left(d/D\right)\cos\alpha \right]$
Bearing roller (BSF)	$\frac{1}{2}f_{r}\left\{1-\left[{\left(d/D\right)}^{2}\cos^{2}\alpha \right]\right\}D/d$
Bearing outer race (BPSO)	$\left(N/2\right)f_{r}\left[1-\left(d/D\right)\cos\alpha \right]$
Bearing inner race (BPFL)	$\left(N/2\right)f_{r}\left[1+\left(d/D\right)\cos\alpha \right]$

**Table 4:** Index comparison table.

Mehtod	ECI	OI	RMSE
TVF–CEITDAN	0.9389	0.0067	0.0932
CEEMDAN	0.5234	0.5346	0.3756
ITD	0.5625	0.3795	0.3472

## Data Availability

Data sharing not applicable.
